# Quercetin Protects Ethanol-Induced Hepatocyte Pyroptosis via Scavenging Mitochondrial ROS and Promoting PGC-1*α*-Regulated Mitochondrial Homeostasis in L02 Cells

**DOI:** 10.1155/2022/4591134

**Published:** 2022-07-16

**Authors:** Xingtao Zhao, Cheng Wang, Shu Dai, Yanfang Liu, Fang Zhang, Cheng Peng, Yunxia Li

**Affiliations:** ^1^State Key Laboratory of Southwestern Chinese Medicine Resources, Ministry of Education, Chengdu 611137, China; ^2^School of Pharmacy, Chengdu University of Traditional Chinese Medicine, Chengdu 611137, China

## Abstract

Alcoholic liver disease (ALD) is a multifaceted process that involves excessive lipid, reactive oxygen species (ROS) production, unbalanced mitochondrial homeostasis, and ultimate cell death. Quercetin is a dietary phytochemical presented in various fruits and vegetables, which has anti-inflammatory and antioxidant effects. According to recent advances in pharmanutritional management, the effects of quercetin on various mitochondrial processes have attracted attention. In the study, we explored whether quercetin could attenuate ethanol-induced hepatocyte pyroptosis by maintaining mitochondrial homeostasis and studied its hepatoprotective effect and the underlying mechanism. We chose L02 cells to establish an *in vitro* model with ethanol-induced hepatocyte pyroptosis. Then, the cells at approximately 80% confluence were treated with quercetin (80, 40, and 20 *μ*M). The cell viability (CCK-8) was used to investigate the effect of quercetin on ethanol-induced L02 cell proliferation. Relative assay kits were used to measure oxidative stress index (OSI = TOS/TAS), lipid peroxidation (LPO) release, and mitochondrial membrane potential (*δ*Ψ*m*). The morphology of mitochondria was characterized by transmission electron microscopy- (TEM-) based analysis. Mitochondrial dynamics (Mito Tracker Green), mitROS (MitoSOX Red Mitochondrial Superoxide) production, and nuclear DNA (nDNA) damage (*γ*H2AX) markers were detected by immunofluorescence. The mRNA levels of mitochondrial function (including mitochondrial DNA (mtDNA) transcription genes TWNK, MTCO1, and MFND) and pyroptosis-related genes were detected by RT-qPCR, and the protein levels of NLRP3, ASC, caspase1, cleaved-caspase1, IL-18, IL-1*β*, and GSDMD-N were detected by western blot. The results showed that quercetin treatment downregulated redox status, lipid droplets, and LPO release, restored damaged mitochondrial membrane potential, and repaired mtDNA damage, PGC-1*α* nuclear transfer, and mitochondrial dynamics. The gene and protein expressions of NLRP3, ASC, cleaved-caspase1, IL-18, IL-1*β*, and GSDMD-N were decreased, which effectively inhibited cell pyroptosis. Therefore, the results indicated that quercetin protected ethanol-induced hepatocyte pyroptosis via scavenging mitROS and promoting PGC-1*α*-mediated mitochondrial homeostasis in L02 cells.

## 1. Introduction

Excessive intake of alcohol has been considered the third largest risk factors threatening human health, and per capita alcohol consumption has increased from 5.9 L to 6.5 L over the past years and is estimated to rise to 7.6 L by 2030 [1]. Excessive alcohol intake may result in alcoholic liver disease (ALD) which has become the leading cause of liver damage after viral hepatitis [2] and has been reported to cause approximately 500,000 deaths per year in Europe [3]. Therefore, the prevention and treatment of ALD have far-reaching significance for maintaining human health. At present, the main treatment medication for ALD is antioxidant and anti-inflammatory drugs, such as corticosteroids, glutathione, hexanone, theobromine, colchicine, and S-adenosine methionine, but these treatments have side effects, including kidney damage and jaundice [4, 5]. Therefore, finding a safer and more effective treatment for ALD treatments is urgently needed [6].

ALD induced by alcohol is a multifaceted process and unbalanced mitochondrial homeostasis. Mitochondria, as highly dynamic organelles, provide most ATP through oxidative phosphorylation to maintain various metabolisms and energy requirements of cells. Studies have revealed that ethanol exposure resulted in imbalance of fusion/fission balance and an immediate change in mitochondrial shape both *in vivo* and *in vitro* [7, 8]. Cytochrome P450 2E1 (CYP2E1), a unique P450 enzyme expressed in hepatocytes, has high catalytic activity for ethanol [9] and results in excessive lipid, ROS production, mitochondrial homeostasis dysfunction, and ultimate hepatocyte pyroptosis [10].

ROS are mainly produced by mitochondria and can mediate various dysfunction of mitochondria, including mitochondrial dynamics, mitochondrial dysfunction, and even pyroptosis. In addition, it is also important in all regulators of inflammatory pathways [11]. Pyroptosis is a new dissolved programmed cell death caused by inflammasome. The nod-like receptor (NLR) family pyrin domain-containing 3 (NLRP3) converts precaspase1 to cleaved-caspase1, which drives the cleavage of gasdermin D (GSDMD) at D275 (numbering after human GSDMD) into N-termini [12]. Upon cleavage, the N-terminus of GSDMD (GSDMD-N) forms a transmembrane pore that releases cytokines such as mature cytokine interleukin-1*β*/18 (IL-1*β*/18), eventually resulting in strong inflammation and cell death [13]. Alcohol induction has also been found to promote NLRP3 inflammasome activation in patients with alcoholic hepatitis, thereby inducing hepatocyte pyroptosis [14]. Moreover, classic caspase1 pathway-dependent hepatocyte pyroptosis has been detected in L02 cells treated with high-concentration ethanol [15]. However, the relationship between mitochondrial homeostasis and pyroptosis remains to be further studied.

Quercetin, a dietary phytochemical presented in various fruits and vegetables, has the ability to scavenge ROS and reduce lipid peroxidation due to its ion chelating and iron stabilizing effect [16]. Currently, it is considered to be related to mitochondrial biogenesis, mitochondrial membrane potential, redox states within mitochondria, and cell death. In high-fat (HF) diet-induced obese mice, quercetin protected mitochondria and counteracted different prooxidant agents [17]. Quercetin upregulated mtDNA content and enhanced the expression of mitochondrial fusion protein 1 (Mfn1) and Mfn2 proteins [18]. Moreover, quercetin possessed a protective effect on macrophage pyroptosis [19]. However, it has not been reported whether quercetin could inhibit pyroptosis in ethanol-induced hepatocyte injury.

## 2. Materials and Methods

### 2.1. Materials

All the experimental materials were shown in Table 1.

### 2.2. Cell Culture

L02 cells (human normal liver cells), obtained from iCell Bio-technology Co., Ltd. (iCell-h054) (Shanghai, China), were cultured in Dulbecco's modified Eagle medium (DMEM) supplemented containing 10% Gibco fetal bovine serum (FBS) (Gibco, Grand Island, NY, United State) and 1% antibiotics (penicillin and streptomycin) at 37°C in a 5% CO_2_ humidified incubator. L02 cells were randomly divided into five groups: (1) the control group, (2) the ethanol (200 mM) model group, (3) ethanol (200 mM)+quercetin (80 *μ*M), (4) ethanol (200 mM)+quercetin (40 *μ*M), and (5) ethanol (200 mM)+quercetin (20 *μ*M). In brief, L02 cells at approximately 80% confluence were treated with quercetin (80, 40, and 20 *μ*M) for 24 h after pretreated with ethanol (200 mM) for 24 h. To prevent ethanol evaporation during exposure, each culture dish was tightly wrapped with parafilm.

### 2.3. Cell Proliferation Assay

L02 cells were cultured in 96-well plates. Different concentrations of ethanol and quercetin were added, each group was set up with 6 duplicate wells, and a group of solvent control group was reserved. After 24 h, the cell density reached 80%, incubated with 10 *μ*l of CCK-8 reagent for 1 h and detected the optical density (OD) value. The absorbance of untreated cells was regarded as 100% of cell survival.

(1) $$\begin{array}{c} \text{Cell viability}=\frac{\text{Treated viable cells}-\text{bank cells}}{\text{Control viable cells}-\text{blank cells}}\times 100\%. \end{array}$$

### 2.4. Oil-Red O Staining

After the experiment, the culture medium was removed, washed with phosphate-buffered saline (PBS) for 3 times, fixed with 4% paraformaldehyde for 30 min to dilute the oil red storage solution (oil red : deionized water = 3 : 2), and filtered with filter paper and heated at 37°C. After dyeing for 10 min, the added dye liquid covered the bottom, and the dye could be absorbed. Then, the dye was rinsed with 60% isopropanol for 1-2 min, and the excess dye was washed with PBS. The field of view was randomly selected under the light microscope (Leica, German) of the glycerin sealant, the dyeing situation was observed, and the image was taken for analysis by ImageJ software.

### 2.5. Nile Red Staining

After the experiment, the cultured cells were fixed with 4% paraformaldehyde for 20 min. After immobilization, cells were stained with Nile Red for 15 min. After wash with PBS, the nucleus was then stained with DAPI for 5 min. The field of view was randomly selected under a fluorescent inverted microscope Confocal Imaging System (Leica Microsystems).

### 2.6. Biochemical Index Determination

L02 cells were cultured in 96-well plates. After the experiment, the supernatant was collected to determine the subcellular redox status by TAS, TOS ELISA kits, and lipid peroxide level by the LPO ELISA kit. The final results were presented as the average of the three independent measurements.

### 2.7. Mitochondrial Membrane Potential (*δ*Ψ*m*) Detection

After treatments, the cells were incubated with a JC-1 staining solution for 20 min at 37°C and washed off with PBS, and the images were obtained by a fluorescent inverted microscope Confocal Imaging System (Leica Microsystems) with randomly selected fields of vision, respectively (*n* = 6) and analyzed by ImageJ software.

### 2.8. Mitochondrial Dynamics and Mitochondrial ROS (MitROS) Content Detection

Cells were incubated with 150 nM Mito-Tracker green for 30 min and then stained with DAPI for 5 min at 37°C. And cells were incubated with 5 *μ*M MitoSOX Red in the dark for 10 min at 37°C. Afterward, cells were washed with PBS for 3 times to remove the dye. Subsequently, cells were analyzed by flow cytometry. Moreover, the images were obtained by a fluorescent inverted microscope Confocal Imaging System (Leica Microsystems) with randomly selected fields of vision, respectively (*n* = 6), and analyzed by ImageJ software.

### 2.9. Immunofluorescence Analysis

After the experiment, the cells were washed with PBS and fixed with 4% formaldehyde for 5 min. Then, the cells were permeated with 0.1% Triton X-100 at room temperature for 10 min. Then, it was sealed with 5% BSA for 1 h and incubated overnight with monoclonal PGC-1*α* and *γ*H2AX antibody solution (diluted 1 : 100) at 4°C. Then, it was diluted with Goat Anti-Rabbit IgG (H+L)-Cy3 secondary antibody (diluted 1 : 500) were incubated at room temperature for 1 h. After washing with PBS, the nucleus was then stained with DAPI for 5 min and mitochondria was then stained with Mito-Tracker green for 30 min. The images were obtained by a fluorescent inverted microscope Confocal Imaging System (Leica Microsystems) with randomly selected fields of vision.

### 2.10. Quantitative Real-Time PCR (RT-qPCR) Analysis

Total RNA/DNA was extracted by animal total RNA/DNA isolation kit. After the concentration measurement, Master Premix for first-strand complementary DNA (cDNA) synthesis at the conditions of 42°C for 15 min and 85°C for 5 min according to the Real-Time kit's instructions. The RT-qPCR conditions were performed at 95°C for 3 min, 40 cycles of 95°C for 10 s, and 65°C for 30 s. Each experiment was conducted with three separate biological samples and the formula of 2^-*ΔΔ*Ct^ was used to calculated relative mRNA expression levels of the target genes. All primer sequences were listed in Table 2.

### 2.11. Protein Extraction and Western Blot (WB) Analysis

Cells were lysed with RIPA lysis buffer: protein phosphatase inhibitor : PMSF : protein mixing enzyme inhibitor = 100 : 1 : 1 : 1 and crushed by ultrasonic cell crushing apparatus for 3 min and centrifuged at 13400 g for 10 min in 4°C. Then, the BCA kit was used to detect the protein concentration, and then, protein concentration was adjusted to be consistent and then probed with anti-Bcl2, Bax, caspase3, cleaved-caspase3, IL-18, IL-1*β*, NLRP3, ASC, caspase1, cleaved-caspase1, and GAPDH (diluted 1 : 1000). As an internal reference, the signals were recorded using a Chemidoc imaging system (Tanon, Shanghai, China) and analyzed using ImageJ analysis software.

### 2.12. Transmission Electron Microscopy (TEM) Analysis

The cells after treatments were prefixed with 2% glutaraldehyde and fixed in 1% osmium tetroxide. Next, the samples were dehydrated in ethanol containing 3% uranyl acetate, embedded in epoxy resin and propylene oxide overnight, and polymerized into 70 nm thick slices and stained with lead citrate, using H-7650 transmission electron microscopy (Hitachi) H-7650) test sections independently invited two uninformed pathologists to quantify each section.

## 3. Statistical Analysis

All experimental data were presented by mean ± standard deviation (S.D.) and analyzed by statistical software SPSS 25.0. *p* < 0.05 was regarded as a significant difference and differences among the groups were evaluated statistically using one-way ANOVA.

## 4. Results

### 4.1. Quercetin Inhibited CYP2E1 Activity to Alleviate High-Concentration Ethanol-Induced Hepatocyte Oxidative Stress and Lipid Peroxidation

**Figure 1:**
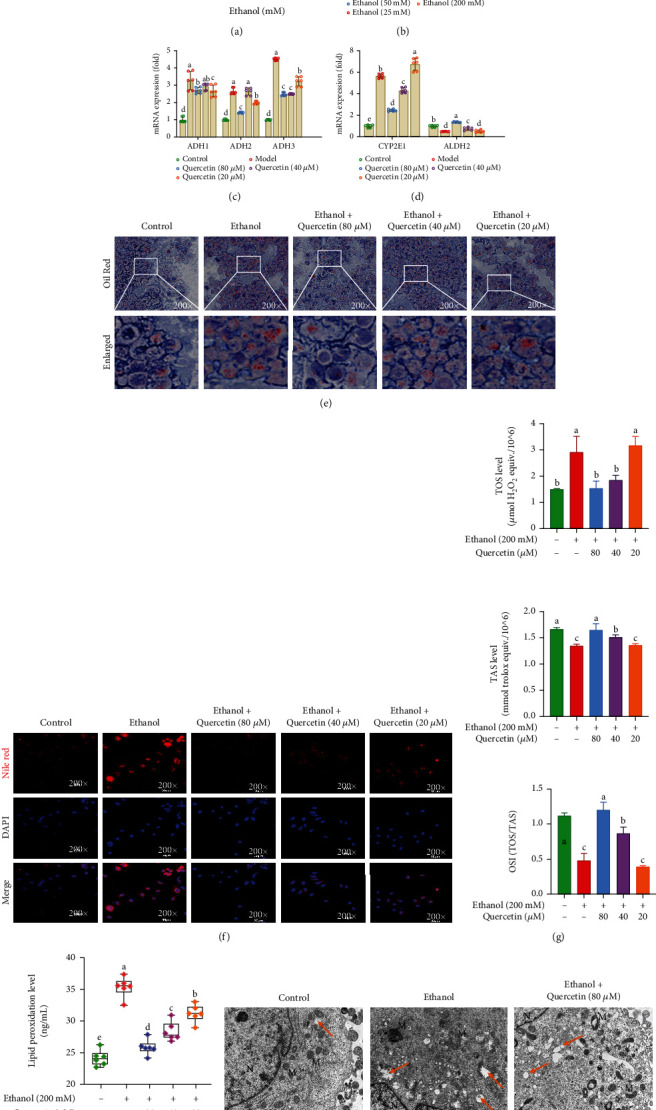
Hepatoprotective effects of quercetin against ethanol exposure. (a) Effects of different concentrations of ethanol on cell viability treated with different concentrations of ethanol for 24 h. (b) Quantitation of levels of ADH and CYP2E1 in L02 cells treated with different concentrations of ethanol for 24 h. (c, d) Quantitation of levels of ADH, CYP2E1, and ALDH2 treated with different concentrations of ethanol for 24 h. (e) Representative images of L02 cells and microphotograph of Oil-Red O staining (200x magnification). (f) Representative immunofluorescent images costained with Nile Red (red), DAPI (blue), and both channels merged. The graphs (down panel) show the fluorescence intensity profiles in two fluorescence channels along the arrow (200x magnification). (g) Determination of TAS, TOS levels, and OSI = TOS/TAS. (h) Determination of LPO level. (i) Representative TEM images of mitochondrial morphology and ultrastructure in L02 cells with boxed areas enlarged. M: mitochondria; N: nucleus; lipid drops (orange arrow) in all groups. Magnification is shown (scale bar, 2.0 *μ*m). Data are expressed as mean ± SD (*n* = 6). Different subscript letters indicate significant differences among the groups (*p* < 0.05).

To investigate the hepatoprotective potential of quercetin, L02 cell line, an ideal cell line widely adopted to research drug toxicity and liver function in *vitro*, was chosen in the current study. Firstly, we identified the most suitable modeling concentration of ethanol by incubating L02 cells with different concentrations of ethanol (25, 50, 100, 200, and 300 mM) for 24 h. With the increase of ethanol concentration (100-300 mM), the CCK-8 results showed that the cell viability of L02 cells decreased significantly (*p* < 0.05). When the ethanol concentration was greater than 200 mM, the cell viability was less than 70% (Figure 1(a)). In addition, with the increase of ethanol concentrations (25-100 mM) for 24 h, the mRNA expression of ADH (ADH1, ADH2, and ADH3) increased significantly (*p* < 0.05), while the expression of CYP2E1 dominating alcohol metabolism did not change until the ethanol concentrations reached to 200 mM (*p* < 0.05) (Figure 1(b)). Interestingly, quercetin effectively reduced the activities of CYP2E1 and enhanced ALDH2 (Figures 1(b) and 1(d)), while did not change ADH significantly (Figure 1(c)), suggesting that the inhibition of CYP2E1 by quercetin may represent a novel therapeutic approach for minimizing the ethanol-induced CYP2E1 enzyme activity. Thus, 200 mM ethanol (24 h) was used in the subsequent experiments. Massive lipid droplets were dramatically reduced by quercetin in ethanol-induced hepatocytes through Oil Red O and Nile Red staining (especially 80 *μ*M quercetin) (Figures 1(e) and 1(f)). Moreover, the same results were also observed by TEM (Figure 1(i)). In the model group, the level of TAS was significantly decreased, whereas the levels of TOS and OSI were significantly increased (Figure 1(g)). However, quercetin treatment significantly reversed the effect of alcohol and the effect of 80 *μ*M quercetin was the most obvious. The release of LPO was significantly elevated in ethanol-induced hepatocyte injury. Quercetin could effectively decrease the release of LPO (*p* < 0.05), and the effect of quercetin was better with the increase of concentration (Figure 1(h)). Collectively, quercetin alleviated CYP2E1-mediated hepatic oxidative stress and lipid peroxidation, thus preventing alcoholic liver injury.

### 4.2. Quercetin Reduced mitROS Generation and Maintained Mitochondrial Dynamics in Ethanol-Induced Hepatocytes

**Figure 2:**
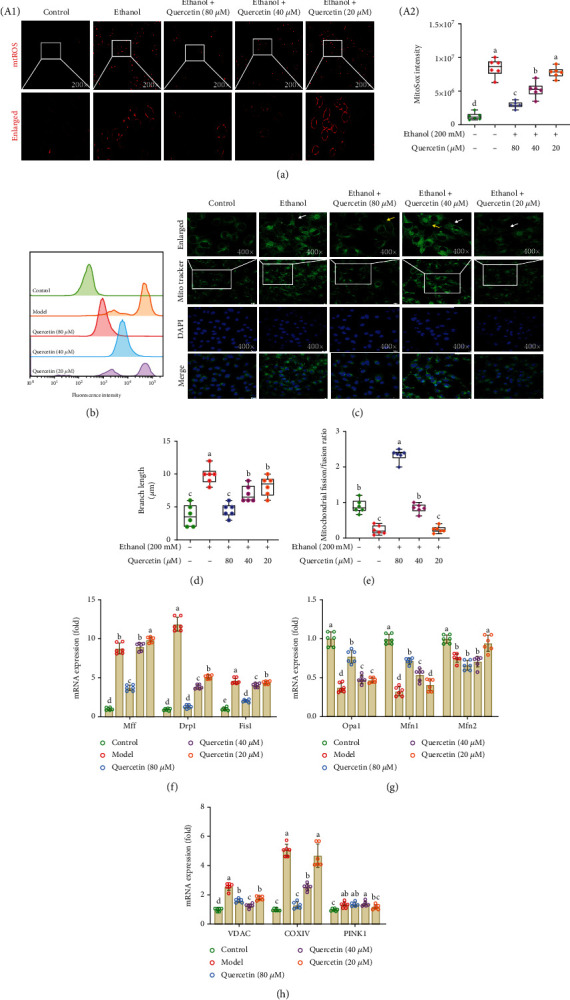
Quercetin reduced ethanol-induced mitROS generation and maintained mitochondrial dynamics. (a) Images of MitoSox Red staining for mitochondrial superoxide (200x magnification), the graphs (down panel) show the fluorescence intensity profiles in two fluorescence channels along the arrow. (b) Quantitative analysis of fluorescence intensity and mitROS generation by flow cytometry. (c) Representative immunofluorescent images co-stained with Mito-Tracker (green), DAPI (blue), and both channels merged (400× magnification). The graphs (up panel) show the fluorescence intensity profiles in two fluorescence channels along the arrow, and the white arrows represent fission; the yellow arrowheads show fusion. (d, e) Quantitative analysis of mitochondrial fission and fusion ratio and fission length. (f–h) The mRNA expression of mitochondrial function genes (VDAC, COXIV, and PINK1), mitochondrial fission genes (Mff, Drp1, and Fis1) and fusion genes (Opa1, Mfn1, and Mfn2). Data are expressed as mean ± SD (*n* = 6). Different subscript letters indicate significant differences among the groups (*p* < 0.05).

Mitochondria are known for their active dynamics, and mitochondrial CYP2E1 acts as an activity generator of mitROS, whose release inhibits electron transfer along the respiratory chain, exacerbating mitochondrial dysfunction [20–22]. TEM (Figure 1(I)) was conducted to observe liver mitochondrial changes. Cells in the control group showed relatively normal mitochondria with intact membranes and cristae. However, ethanol treatment caused various degenerative changes including abnormal shape swelling, perinuclear aggregation with a dark matrix, fragmentation, and destruction of intima. Quercetin ameliorated the damage, with slight swelling in mitochondria. Compared with the control group, massive mitROS were produced in hepatocytes after ethanol treatment, while quercetin reduced mitROS production, shown by both immunofluorescence and flow cytometry analysis (*p* < 0.05) and the effects of 80 and 40 *μ*M quercetin were obvious. (Figures 2(a) and 2(b)). Mitochondria are extremely dynamic organelles, constantly undergoing antagonistic processes of fission and fusion in maintaining mitochondrial function and cellular homeostasis. Recently, studies have shown that high-concentration ethanol exposure can cause mitochondrial fission and fusion imbalance in mice. We firstly detected mitochondrial fusion and fission through Mito-Tracker Green staining (Figure 2(c)), and the morphology changes of mitochondria were observed by confocal microscope. These were length or aspect ratio (AR, the ratio between the major and minor axis of the ellipse equivalent to the mitochondrion) and degree of branching or form factor (FF, defined as (*Pm*2)/(4*πAm*), where *Pm* is the length of mitochondrial outline and *Am* is the area of mitochondrion). Since the mitochondria within a cell were often either oval or fragmented, we classified the cells as mitochondria fusion based on FF < 1 and the shape is more uniformly spherical, and mitochondrial fission based on AR > 1 and the majority (>70%) of fragmented mitochondria as previous reported [23, 24]. And our quantitative determination revealed that the length of mitochondria was significantly reduced after quercetin treatment compared with ethanol group (*p* < 0.05) (Figures 2(d) and 2(e)). In addition, the expression of related genes was determined by RT-qPCR (Figures 2(f)–2(h)), which indicated that 80 *μ*M quercetin increased the mRNA levels of fusion genes (Opa1 and Mfn1) and inhibited the mRNA levels of fission genes (Mff, Drp1, and Fis1) in ethanol-treated hepatocytes (*p* < 0.05). Quercetin reduced Drp1 gene expression in a dose-dependent manner, suggesting that mitochondrial fission may play an important role.

### 4.3. Quercetin Restored Ethanol-Induced Mitochondrial Membrane Potential and Alleviated mtDNA Damage Unrelated to Apoptosis

**Figure 3:**
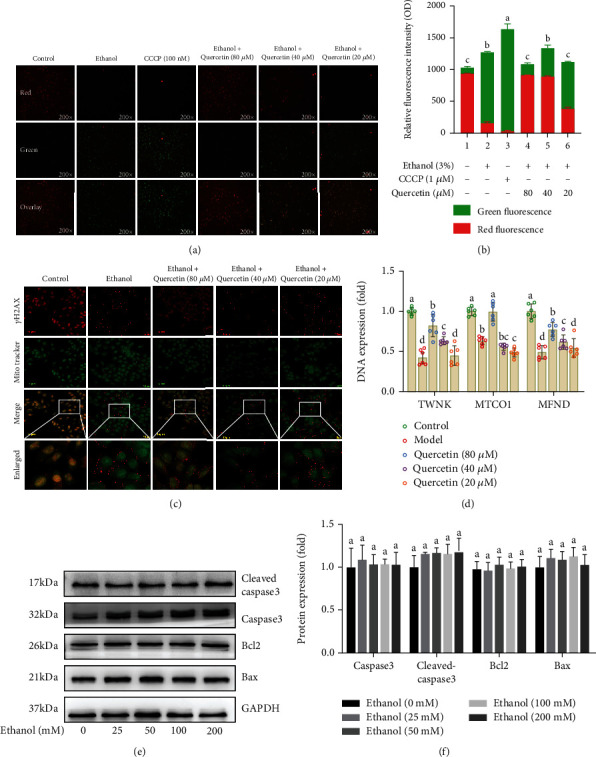
Quercetin restored ethanol-induced mitochondrial membrane potential and alleviated mtDNA damage unrelated to apoptosis (a) Representative immunofluorescent images of JC-1 is visible either as JC-1 monomers (green), JC-1 aggregates (red), and both channels merged (200x magnification), whereas more JC-1 aggregates (red) were seen in quercetin treatment while more JC-1 monomers (green) in ethanol group. (b) Relative fluorescence intensity calculated as red to green ratio. (c) Representative immunofluorescence images co-stained with *γ*H2AX (red), Mito-Tracker (green) and both channels merged (400x magnification). The graphs (down panel) show the fluorescence intensity profiles in two fluorescence channels along the arrow, whereas a weaken fluorescence intensity of *γ*H2AX is seen in quercetin treatment and enhanced by ethanol. (d) The mRNA expression that encodes the enzyme (TWNK, MTCO1, and MFND) responsible for mtDNA transcription levels. (e) Western blot analysis of Bax, Bcl2, caspase3, and cleaved-caspase3 protein abundance. Data are expressed as mean ± SD (*n* = 6). Different subscript letters indicate significant differences among the groups (*p* < 0.05).

Increased ROS activates the opening of mitochondrial permeability transition pores (mPTP), which creates a channel through which mtDNA can be transferred from mitochondria to the cytoplasm, disrupting mitochondrial homeostasis [25]. Mitochondrial membrane potential was detected by JC-1 kit. In normal cells, the mitochondrial membrane potential is high, and the dye aggregates into polymers in the mitochondrial matrix, forming red fluorescent aggregates (JC-1 aggregates). The change of mitochondrial membrane potential prevents the accumulation of JC-1. Therefore, the dye exists as a monomer and produces green fluorescence (JC-1 monomers), and CCCP (1 *μ*M) was used as a positive control to induce the decrease of mitochondrial membrane potential. The results (Figures 3(a) and 3(b)) showed that the fluorescence with ethanol treatment changed from red to green, mitochondrial membrane potential was decreased, and mitochondrial membrane were certainly damaged (*p* < 0.05). RT-qPCR results also showed the gene that encodes the enzyme (TWNK, MTCO1, and MFND) responsible for mtDNA transcription levels increased under high-concentration ethanol (*p* < 0.05), which was significantly reversed by 80 *μ*M quercetin (Figure 3(d)). In addition, it has been reported that mtDNA damage can induce cell death [26, 27]. Moreover, a change of mtDNA damage resulted in nDNA, the double-strand breaks rapidly leads to Histone-2AX (H2AX) phosphorylation (*γ*H2AX) [28]. Our immunofluorescence results showed that ethanol stimulated *γ*-H2AX formation (red fluorescence) and the spread of *γ*-H2AX reduced after quercetin treatment (Figure 3(c)). However, we detected apoptotic proteins (Bax, Bcl2, caspase3, and cleaved-caspase3) and found no significant changes in the related proteins with the increase of ethanol concentration (Figures 3(e) and 3(f)). These results suggested that apoptosis is not involved in ethanol-induced mitochondrial membrane potential and mtDNA damage and there may be a new pathway of cell death.

### 4.4. Quercetin Alleviated Ethanol-Induced Pyroptosis

**Figure 4:**
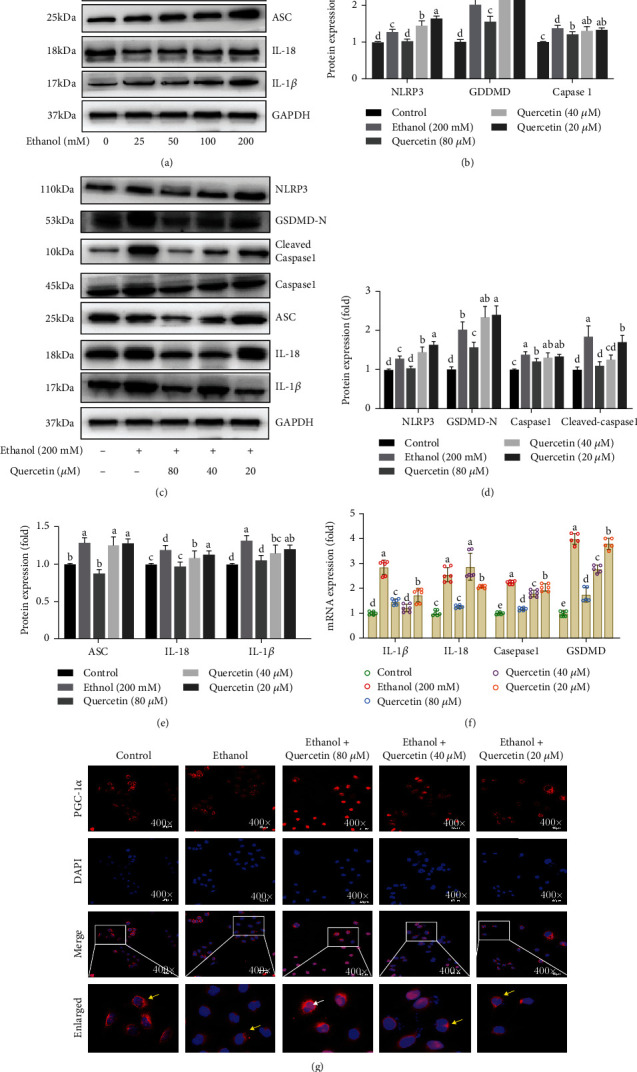
Quercetin alleviated ethanol-induced pyroptosis via the nuclear transfer of PGC1*α*. (a–e) Western blot analysis of NLRP3, caspase1, cleaved-caspase1, GSDMD-N, ASC, IL-18, and IL-1*β* protein abundance. (f) The mRNA expression of IL-1*β*, IL-18, caspase1, and GSDMD genes. (g) Representative immunofluorescent images co-stained with PGC-1*α* (red), DAPI (blue) and both channels merged (400x magnification). The graphs (down panel) show the fluorescence intensity profiles in two fluorescence channels along the arrow and the white arrows represent nucleus PGC-1*α*; the yellow arrowheads show cytoplasm PGC-1*α*, whereas a clear nuclear translocation (white arrow) and shrinkage of PGC-1*α* (red) is seen in quercetin treatment and inhibited by ethanol. Data are expressed as mean ± SD (*n* = 6). Different subscript letters indicate significant differences among the groups (*p* < 0.05).

Mitochondrial oxidative damage leads to a series of inflammatory responses [29]. ROS can act as a triggering factor for the activation of NLRP3 inflammasome and a bonfire or effector molecule leading to pathological processes [11]. Pyroptosis is a caspase1-dependent programmed cell death, known as the canonical inflammasome pathway. Compared with control group, various inflammasome-associated proteins (IL-1*β*, IL-18, cleaved-caspase1, ASC, and GSDMD-N) as well as inflammasome sensors (NLRP3) showed higher levels in ethanol group, while quercetin decreased the activity of NLRP3, ASC, and cleaved-caspase1. And cleaved-caspase1 drives the maturation of proinflammatory cytokine IL-1*β* and IL-18 and the cleavage of GSDMD at D275 (numbering after human GSDMD) into N-termini, eventually resulting in strong inflammation and cell death (Figures 4(c)–4(e)). Consistently, RT-qPCR results revealed similar mRNA expression patterns of NLRP3, GSDMD, ASC, IL-1*β*, and IL-18 (Figure 4(f)), suggesting quercetin protected ethanol-induced hepatocyte pyroptosis. Moreover, we next sought to determine the feasibility of alleviating ethanol-induced pyroptosis by PGC-1*α*. It is a transcriptional coactivator and acts as a crucial factor in transcription of nuclear-encoded mitochondrial genes [30]. Immunofluorescence results showed that there was a substantial decline in PGC-1*α* nuclear translocation when exposed to high concentration ethanol, while treatment with quercetin induced PGC-1*α* nuclear translocation efficiently (Figure 4(g)), suggesting that quercetin-mediated nuclear transfer of PGC-1*α* may play an important role in alcohol-induced hepatic pyroptosis.

## 5. Discussion and Conclusion

Quercetin, a bioactive secondary metabolite, holds incredible importance in terms of bioactivities, which has been proved by *in vivo* and *in vitro* studies [16]. Further, corresponding to the growing population and global demand for fresh fruits and vegetables, a paradigm shift and focus is laid towards exploring the mechanistic role of quercetin in hepatic pathologies. Our results indicated that quercetin protected ethanol-induced hepatocyte pyroptosis via scavenging mitROS and promoting PGC-1*α*-mediated mitochondrial dynamics to alleviate hepatocyte pyroptosis in L02 cells. And it rendered quercetin an ideal phytochemical that can provide protective benefits against ALD.

Since hepatocytes are the prime liver parenchymal cells, L02 cell line, as an ideal cell line was incubated with ethanol to establish an in vitro model of ALD in our study [31]. In the liver, alcohol dehydrogenase (ADH) and CYP2E1 are the major oxidative pathways of alcohol metabolism, with a secondary pathway through peroxisome catalase [20]. CYP2E1, a major contributor to ROS generation, is induced by chronic/excessive alcohol consumption and progresses to an advanced disease stage [32]. With the increase of ethanol concentrations (25-100 mM) for 24 h, the mRNA expression of ADH (ADH1, ADH2, and ADH3) increased significantly, while the expression of CYP2E1 dominating alcohol metabolism did not change until the ethanol concentration reached to 200 mM. Interestingly, quercetin effectively reduced the activities of CYP2E1 and enhanced ALDH2, while did not change ADH significantly, suggesting that the inhibition of CYP2E1 by quercetin may represent a novel therapeutic approach for minimizing the ethanol-induced CYP2E1 enzyme activity, which results the hepatotoxicity of ethanol. Thus, a large dose of ethanol (200 mM) was used to expose to L02 cells.

Prior research showed that mitochondrial CYP2E1 had very high NADPH oxidase activity, which can reduce ATP production in mitochondrial electron transport system and increase electron leakage on MRC during chronic drinking [33]. Our research also showed that mitochondrial CYP2E1 played an important role in high acute ethanol exposure. Mitochondria, as highly dynamic organelles, provide most adenosine triphosphate (ATP) production through oxidative phosphorylation (OXPHOS) for normal function [34]. The OXPHOS system consists of five polysubunit complexes in the inner mitochondrial membrane (IMM), consisting of nDNA and mtDNA coding [35]. IMM CYP2E1 acts as a ROS activity generator [33], and mtDNA is located in the inner membrane and not protected by histones, so it is more vulnerable to ROS attack than nDNA, leading to mitochondrial dysfunction [36]. mitROS can inhibit the synthesis of related protein subunits responsible for encoding mtDNA, potentially disrupting the cycle of oxidative phosphorylation, which in turn releases more mitROS and continues to damage mtDNA, thereby creating a vicious cycle of biological energy disorders to further affect the survival of cells and tissues, and finally lead to the occurrence of cell death [37–39]. Similarly, the genes encoding the enzyme (TWNK, MTCO1, and MFND) responsible for mtDNA transcription levels were observed in our experiment. JC-1 experiment showed that the loss of mtDNA leaded to the change of mitochondrial membrane potential. Apparently, quercetin alleviated the collapse of the mitochondrial membrane potential to maintain mitochondrial homeostasis.

In addition, inhibition of mitochondrial dynamics also affects the elimination of irreparable mtDNA damage and the transmission of mtDNA mutations [40]. Mitochondrial dynamics is achieved through continuous crest remodeling and the fission and fusion of mitochondrial membranes. Mitochondrial fusion is a complex process involving the connection of the outer and inner membranes of mitochondria, which facilitates the communication between mitochondria and their host cell to maintain cell homeostasis. Mitochondrial outer membrane (OMM) is fused with Mfn1 and Mfn2, and then, IMM is fused with mitochondrial kinetic protein-like GTPase (OPA1) [41]. Mitochondrial fission is triggered by the contraction of endoplasmic reticulum (ER) membrane. The receptors on OMM include fission 1 protein (Fis1), mitochondrial fission factor (Mff), and dynamin-related protein 1 (Drp1), which are involved in controlling the number and distribution of mitochondria and respond to changes in cellular energy requirements [40]. Our study found that quercetin inhibited the expressions of mitochondrial fusion genes including Mfn1, Mfn2, and OPA1, as well as increased fission genes expressions, and and the most significant change was Drp1, which played an important role in mediating mitophagy.

Previous studies indicated that unbalanced mitochondrial homeostasis and ROS generation are often associated with a novel type of cell fate, pyroptosis, which is different from previous apoptosis because of depending on the NLRP3/cleaved-caspase1/GSDMD-N activation [42]. In the current study, we demonstrated that it did not restore cell viability when simply reversing apoptosis in high-concentration ethanol-treated hepatocytes, which suggested that other kinds of cell fates such as pyroptosis may be involved. Therefore, we further investigated whether quercetin could alleviate ALD by inhibiting pyroptosis. As an intracellular target of ROS, NLRP3 inflammasome plays a key role in alcohol-induced acute liver injury [43]. Our results found that the inactivation of NLRP3 inflammasome was needed for quercetin to inhibit caspase1 activation in ethanol-induced hepatocytes. Here, we firstly confirmed the curative effect of quercetin to reduce NLRP3 inflammasome-cleaved-caspase1/ASC and GSDMD-N activation and decrease IL-1*β* and IL-18 secretions in LO2 cells, alleviating hepatocyte pyroptosis in ALD. Similarly, PGC-1*α*, a key mitochondrial biogenesis regulator, modulates NLRP3 inflammasome and attenuates hepatocyte pyroptosis in ALD [15]. In the current study, quercetin promoted the nuclear translocation of PGC-1*α*. Consistent with anterior studies, our data showed that NLRP3 served as a downstream event of PGC-1*α* to reduce the excessive ROS accumulation in impaired hepatocytes, which might be the key mechanism of quercetin in relieving hepatocyte pyroptosis. However, how quercetin specifically affects PGC-1*α* nuclear translocation and whether PGC-1*α* directly activated transcription of GSDMD-N to induce pyroptosis remain to be further studied.

**Figure 5:**
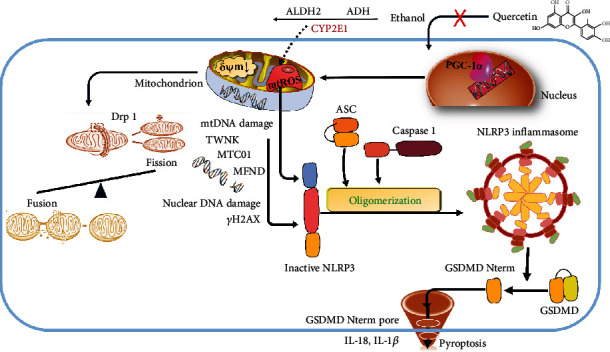
The mechanism of quercetin protected ethanol-induced hepatocyte pyroptosis.

Taken together (Figure 5), it was demonstrated that quercetin protected ethanol-induced hepatocyte pyroptosis via scavenging mitochondrial ROS and maintaining mitochondrial homeostasis. Mechanistically, quercetin promoted PGC-1*α* transcription and subsequently restored mitochondrial function and maintained mitochondrial dynamic balance, which ultimately protected hepatocytes from pyroptosis. The above results suggested that quercetin, as a novel phytochemical supplement, had the potential in antihepatic injury at least in part by remodeling PGC-1*α*-mediated mitochondrial dynamics to alleviate hepatocyte pyroptosis.

## Figures and Tables

**Table 1:** Information about experimental materials.

Raw materials	Biotechnology Co., Ltd	ID
Quercetin (purity > 99.00%)	MUST, Chengdu, China	117395
Cell Counting Kit-8	G-clone, Beijing, China	CC1410-500
Ethanol and other organic reagents	Kelong, Guangdong, China	
Total antioxidant status (TAS) ELISA kit	Elabscience, Wuhan, China	E-BC-K801
Total oxidant status (TOS) ELISA kit	Elabscience, Wuhan, China	E-BC-K802
LPO ELISA kit	MeiMian, Jiangsu, China	MM-1378H1
Oil red storage solution	Solar, Wuhan, China	G1015
Nile Red staining solution	Solar, Wuhan, China	N8440
JC-1 Mitochondrial Membrane Potential Assay	Solar, Wuhan, China	G1515
*γ*h2AX antibody	Solar, Wuhan, China	GB111841
MitoSOX Red Mitochondrial Superoxide	Yeasen, Shanghai, China	40778ES50
DAPI staining solution	Yeasen, Shanghai, China	40728ES03
Mito Tracker Green	KeyGEN, Jiangsu, China	KGMP007
IL-18 antibody	Affinity, Cincinnati, OH, USA	DF6252
IL-1*β* antibody	Affinity, Cincinnati, OH, USA	AF5103
Caspase 1 antibody	Affinity, Cincinnati, OH, USA	AF5418
Cleaved-caspase 1 (Ala317), p10 antibody	Affinity, Cincinnati, OH, USA	AF4022
NLRP3 antibody	Affinity, Cincinnati, OH, USA	DF7438
GSDMD-N-terminal antibody	Affinity, Cincinnati, OH, USA	DF12275
GAPDH antibody	Affinity, Cincinnati, OH, USA	AF0911
Cleaved-aspase3 (Asp175), p17 antibody	Affinity, Cincinnati, OH, USA	AF7022
Bcl2 antibody	Abmart, Shanghai, China	T40056
Bax antibody	Abmart, Shanghai, China	TP51006
Caspase3 antibody	Abmart, Shanghai, China	T55051
ASC/TMS1	ZEN, Chengdu, China	340097
PCG-1*α*	ZEN, Chengdu, China	862041
Goat Anti-Rabbit IgG(H+L) HRP secondary antibody	Multi science, Hangzhou, China	GAR007
Goat Anti-Rabbit IgG (H+L)-Cy3 secondary antibody	ABclonal, Wuhan, China	AS040
Total RNA isolation kit	Foregene, Chengdu, China	RE-03014
Animal tissue DNA isolation kit	Foregene, Chengdu, China	DE-05011
Master Premix for first-strand cDNA synthesis	Foregene, Chengdu, China	RT-01021
Real-Time PCR EasyTM-SYBR Green I	Foregene, Chengdu, China	QP-01011

**Table 2:** The gene primer sequences used for RT-qPCR.

Gene name	Forward primer (5′->3′)	Reverse primer (5′->3′)
*ADH1*	TGGGAAAAGTATCCGTACCATT	AGCTGTTGCTCCAGATCATGT
*ADH2*	GGGCAGAGAAGACAGAAACGA	TTCCTCTCATCAAACTTCTGTCA
*ADH3*	CCCAAACTTGTGGCTGACTTT	ACTCTTTCCAGAGCGAAGCA
*CYP2E1*	GAAGCCTCTCGTTGACCCAA	TGGTGGGATACAGCCAAACC
*ALDH2*	CATGGACGCATCACACAGGG	CTTGCCATTGTCCAGGGTCT
*COXIV*	GGCGGCAGGTGTACATTTTTA	AGTCTTCGCTCTTCACAACA
*VDAC*	CCCAAACTTGTGGCTGACTTT	ACTCTTTCCAGAGCGAAGCA
*PINK1*	AACCGCTTCGACTTTCTGCT	CACTTGATGAACCAGCCCCA
*Mfn1*	TGGCCACATGTAGTTTATGTTTC	TTGCACCTGCTGTAAAAAGGC
*Mfn2*	GGAAGGTGAAGCGCAATGTC	TGCATTCACCTCAGCCATGT
*Opa1*	CTGTGGCCTGGATAGCAGAA	AGACTGGCAGACCTCACTCT
*Drp1*	CCGGAGACCTCTCATTCTGC	TCTGCTTCCACCCCATTTTCT
*Fis1*	TCAGCCCCATCATGTGCTTT	CAGGAGAGGACCAGGAGTGA
*Mff*	CTCTCAGCCAACCACCTCTG	GTGCTGGATTGAGAGCCACT
*TWNK*	CTGGTTGGGGGATGCCTTC	ATTGAAGCCTCCGTTCAGGG
*MFND*	CAACTACGCAAAGGCCCCA	TGATGGTAGATGTGGCGGGT
*MTCO1*	CTATCCGGAATGCCCCGA	GGCATCCATATAGTCACTCCAG
IL-18	TGCAGTCTACACAGCTTCGG	GCAGCCATCTTTATTCCTGCG
IL-1*β*	TGATGGCTTATTACAGTGGCA	CGGAGATTCGTAGCTGGATG
Caspase-1	CCTGCCGTGGTGATAATGTT	TCCACATCACAGGAACAGGC
*GSDMD*	CAGAAGGGACGTGGTGTTCC	AGTTTACGGAAGTCGGCGAG
GAPDH	ACTAGGCGCTCACTGTTCT	CCAATACGACCAAATCCGTTG

## Data Availability

The underlying data of the study can be obtained by contacting the authors if it is reasonable.

## Abbreviations

ADH: Alcohol dehydrogenase
ALD: Alcoholic liver disease
ALDH2: Aldehyde dehydrogenase 2
ATP: Adenosine triphosphate
CYP2E1: Cytochrome P450 2E1
Drp1: Dynamin-related protein 1
ER: Endoplasmic reticulum
Fis1: Fission 1 protein
GSDM: Gasdermin
GSDMD-N: N-terminus of GSDMD
HF: High fat
IL-1*β*/18: Interleukin-1*β*/18
IMM: Inner mitochondrial membrane
Mff: Mitochondrial fission factor
Mfn1: Mitochondrial fusion protein 1
mPTP: Mitochondrial permeability transition pores
mtDNA: Mitochondrial DNA
nDNA: Nuclear DNA
NLRP3: Nod-like receptor (NLR) family pyrin domain-containing 3
OMM: Mitochondrial outer membrane
OPA1: Mitochondrial kinetic protein-like GTPase
OXPHOS: Oxidative phosphorylation
ROS: Reactive oxygen species
TEM: Transmission electron microscopy
*γ*H2AX: Histone-2AX (H2AX) phosphorylation.

## Additional Points

*Institutional Review Board Statement*. The experiments were performed under the approval of the Institutional Animal Care and Use Committee of Chengdu University of Traditional Chinese Medicine (No. SYXK (CHUAN) 2014−128).
